# Eyes say more than we think

**DOI:** 10.3325/cmj.2011.52.578

**Published:** 2011-08

**Authors:** Nika Matovac

It’s finally summer and I’ve finished my third year of medical school, so I am going to have long and carefree holidays. This made wonder about how patients feel during their holidays and whether they can stop thinking about their illness just for a while. From my experience I would say NO; they think about it even more, but what can be done about it?

I remember when holidays were getting closer my dad and I were very excited, but my mum who was dealing with breast cancer wasn’t. She wasn’t able to stay long in the sun because of her radiation therapy, she had problems with her implant, and all together she couldn’t enjoy herself. I remember how I would tell her: “Mum, you are so brave, I would never be able to fall asleep with the knowledge of having something inside me, especially cancer.”

What happens when a patient like my mum goes on holidays? Although I can say that she was doing well in general, I remember that summer was the time I saw her crying the most. She would say that she has to do something that would drag her thoughts from cancer, from doctors, from therapy, and most of all from the idea of her future life, when these therapies will stop and how long she is going to live. Although death is a part of everyone’s life and no one knows how long one is going to live, oncology patients think about such things even more.

This year, I had a course called Psychological Medicine, at which we also learned how to approach and prepare not only patients, but also their families, for their long and tiresome journey to cure. Martin H. Fischer said: “Disease is somatic, suffering because of it is mental.” This quote says how important it is to have a whole team of doctors to guide such patients, their families, and friends. My mum had one million questions for her oncologist about the therapy, about how she is doing, whether there were some newer and better medicines, but also she had one million thoughts that she wanted to share with some psychiatrist. She was afraid about my future (I was only 11 when her breast cancer was diagnosed), she was dealing with problems that go with therapy (hair loss, radical mastectomy), and she was dealing with the idea of dying. All this is not easy and help is needed. Back in 2001, there wasn’t so much information about where to go, who to talk to, and how to deal with the mental part of illness. At that time, cancer was some sort of stigma, which no one wanted to talk about.

I didn’t want my friends to know about my mum, not because I was ashamed of it, but because when I told someone, I saw pity in their eyes and that was the last thing I wanted to see. At the age of 11, I didn’t want do deal with the idea of death. My classmate asked if it was true that my mum had silicone implant instead of a breast. I don’t ask you to understand how I felt then, but just try… I was a child and, as I already said, any kind of illness back then was a stigma, and people associated silicone implants only with breast augmentation. I was standing speechless in front of my classmate and wasn’t able to answer. I think that today we are going ahead, but there is still a lot to do.

If you ask me, everyone who is diagnosed with any kind of malignant tumor should be able to talk to a psychiatrist, who would prepare the family, especially the children, to do deal with the disease. I met a woman who had been diagnosed with breast cancer at a very young age and her children were rather young – her daughter was about five and her son was two. Soon after her surgery, she started receiving chemotherapy and not long after she was bald. Seeing their mum, who had had beautiful long hair, bald was very stressful for the children. Her son was so afraid of her that he started crying every time he was around her. For a woman who was dealing with illness and therapy, it was heartbreaking to see her baby son running away from her in tears.

Patients don’t need only their chemotherapy, radiation, or surgery, they need much more. They need someone to talk to, they need a psychiatrist to go with them through their problems, to advise their children, husbands/wives, and parents what to do, how to react, and how to help. I am thrilled because we have finally started doing something about this. At the end of June 2011, the patients’ association ‘Krijesnice’ published a picture book ‘When Someone Is Very Ill’ intended for children who have a seriously ill family member, to help them accept thoughts and feelings related to the illness. Furthermore, there is also Psychological Assistance Centre ‘Everything for Her!,’ where anyone can come and ask for individual, partnership, or family psychological support, group psychological treatments, or support groups. It also offers workshops on nutrition, physiotherapy, special types of yoga and relaxation techniques, and other anti-stress techniques. All of these programs are well attended and there are more and more users each month. Although this might seem enough, I will say that never is enough. We still need to educate our doctors how to approach such patients and how to communicate bad news.

As a medical student, I see that nowadays doctors are more and more focused on paperwork (not because they want to but because they have to) than on their patients. I have learned that you can learn and find out a lot about your patient just from a simple talk, but who has the time to talk to their patient with 20 more patients waiting outside? Fortunately, we are moving forward and there are many associations where patients can go, find help, or talk with other patients and hear their experience. I am aware that Croatia lacks doctors and that each doctor has many patients each day, but still we have to try to look at the patients rather than act like robots running on a treadmill. We have to be aware that we can cure not only with medicine or surgery, but also with words.

My mum, when I started my third year of medical school, told me: “Nika, just listen to your patients and talk to them, it is really important to have human relationship with them, they are not cases, they are above all people, who have feelings, thoughts, and opinions; who have children, families, and friends and who are scared and need you.”

At the end, once again I would like to point out how important it is to communicate with patients, ask them how their illness affects their families, and how they are dealing mentally with the illness. Also, there might be patients who don’t talk much and these are the patients we should look in the eyes. It is said that the eyes are the mirror of the soul, so only by looking in the eyes we can find out how our patient feels, we can see fear, we can see despair, we can also see happiness and thrill. So keep talking, don’t run away from those who need us, and remember that doctors and patients are human beings with the same needs and the same feelings.

